# Enhancing the use of T cells as a universal preventive measure against H3N2 influenza: Mechanism and implications

**DOI:** 10.1002/hsr2.70004

**Published:** 2024-08-20

**Authors:** Jugal Hiren Bhatt, Nency Kagathara, Kahan Mehta, Maurya Joshi, Mohamed A. Omar

**Affiliations:** ^1^ Department of Internal Medicine GMERS Medical College Gotri Vadodara India; ^2^ Department of Medicine Zydus Medical College Dahod India; ^3^ Faculty of Health Sciences University Way Nairobi Kenya

Dear Editor,

I am writing to bring to your attention a matter of significant global concern—the recurrent outbreaks of Influenza (H3N2) that have emerged in various parts of the world over the past year. These outbreaks not only underscore the virulence and adaptability of the Influenza (H3N2) virus but also shed light on the critical challenges we face in terms of accurate and timely diagnosis, as well as the pressing need for effective vaccination strategies.^1^ Since, the H3N2 subtype of Influenza A is crucial to address due to its rapid antigenic and genetic changes, which impact vaccine efficacy and lead to more severe infections characterized by elevated C‐reactive protein, higher fevers, and increased leukopenia. In this letter, I emphasize diagnostic and vaccination challenges to overcome Influenza (H3N2) outbreaks and the need for global collaboration.^2^, ^3^

T cell‐mediated immunity is essential in fighting Influenza (H3N2). CD4 T cells coordinate the immune response and produce IFN‐γ which is an inhibitor of viral replication and is an important inducer of class 1 MHC, expression and macrophage activation, while CD8 CTLs cells directly eliminate infected cells by binding of Fas in the target cell membrane by the Fas ligand, which is present in the membrane of activated CD8 CTLs^4^ (Figure 1). Leveraging T cell responses is critical for developing effective T cell‐mediated vaccines against H3N2, as they can target specific viral antigens, providing lasting protection.^5^ As per a review, next‐generation influenza vaccines utilizing T cells could address the limitations of current antibody‐based vaccines. T cells offer the potential for broader protection, but challenges include clinical validation, memory cell generation, HLA diversity, and immune escape. More human clinical data is needed to understand T‐cell protection. Standardized methods are required for T‐cell assessment. T‐cell vaccines could enhance pandemic preparedness alongside antibody vaccines, but questions remain about their optimal use and timing. This review bridges T‐cell biology and clinical data, essential for a universal influenza vaccine powered by T‐cells.^6^ A double‐blind trial in Australia assessed a T cell‐inducing vaccine (MVA‐NP + M1) in adults after standard influenza vaccination. Although, the vaccine was well‐tolerated, reduced influenza symptoms and length of virus shedding by inducing antigen‐specific T‐cell response but didn't significantly improve influenza incidence, suggesting the need for alternative strategies.^7^ mRNA vaccines have gained significant attention. A recent study showed that mice vaccinated with H1 and H3 COBRA HA‐encoding mRNA vaccines generated robust antibody responses and were protected against H1N1 and H3N2 influenza, outperforming WT HA‐encoding vaccines.^8^ Additionally, a study on a recombinant Pichinde virus (rPICV) as a viral vector showed that pigs vaccinated with rPICV‐H3 or recombinant HA protein had high antibody titers and were protected from IAV‐S, highlighting rPICV's potential as a vaccine vector.^9^ Sequential immunization of cHA vaccines may present a promising strategy for achieving broad influenza protection by bolstering pre‐existing antibody and cellular responses against conserved HA stalk structures in humans.^10^ It can be complex and costly to measure T cell responses and infrequent clinical outcomes, and thus innovations in clinical trial design are needed for economic reasons.^11^

**Figure 1:**
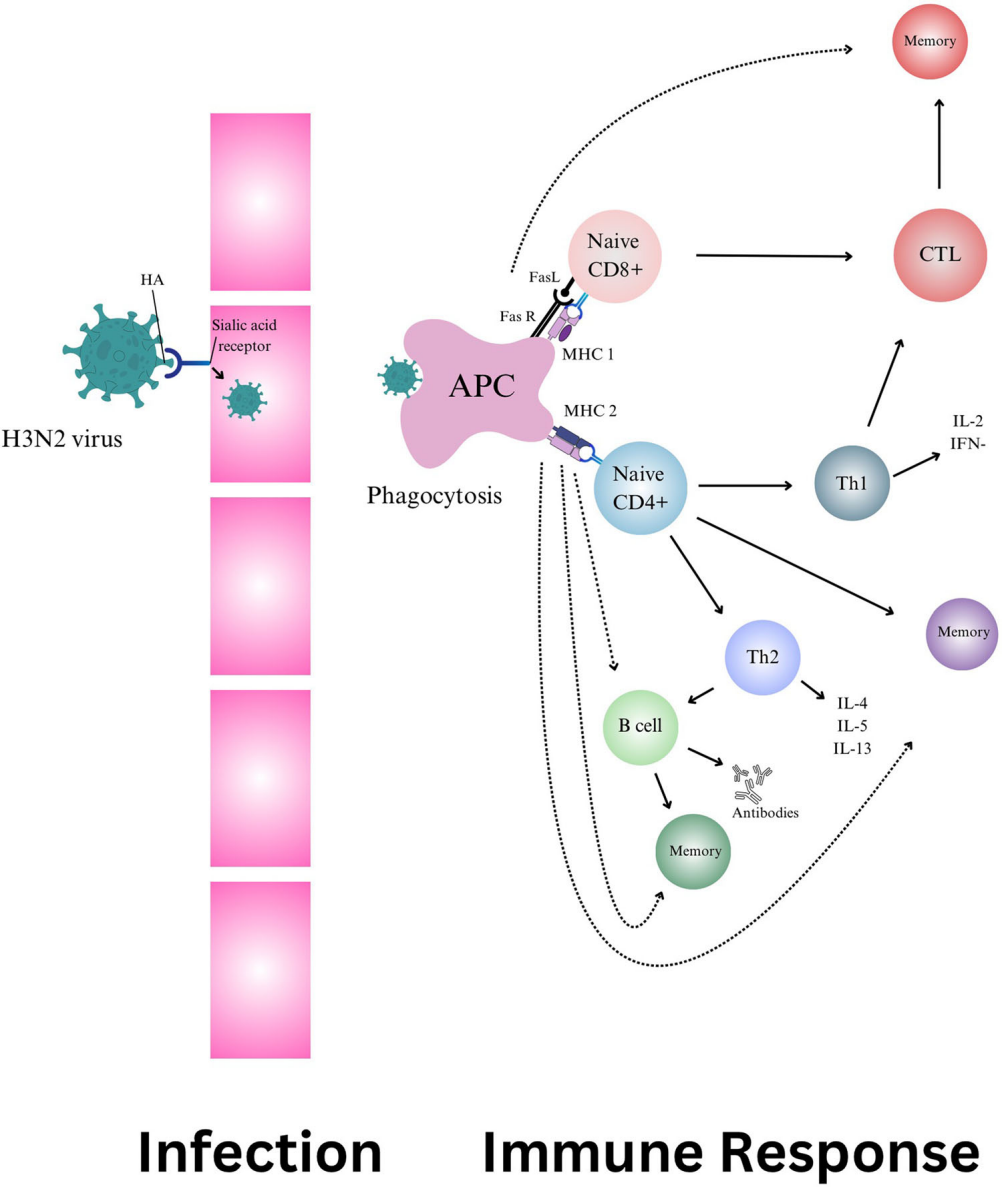
A schematic diagram depicting the process of influenza A (H3N2) virus infection and immune response.

This emphasizes the importance of continued trials and investigations to unlock the full potential of T cell‐based vaccines in enhancing pandemic preparedness alongside traditional antibody‐based vaccines.

Hence continued research on T‐cell‐based vaccines as they offer the potential for broader protection against infectious diseases, including Influenza (H3N2). Standardize methods for assessing T‐cell responses and conduct further clinical studies to explore their efficacy.

Understanding the intricate host interactions during H3N2 infection is crucial for developing effective vaccines. By targeting specific components of the immune response or viral proteins involved in host interactions, researchers can develop interventions that enhance immune recognition, promote viral clearance, and prevent severe disease outcomes.

## AUTHOR CONTRIBUTIONS

**Jugal Hiren Bhatt**: conceptualization; investigation; supervision; writing—review and editing; writing—original draft. **Nency Kagathara**: conceptualization; project administration; writing—review and editing; writing—original draft. **Kahan Mehta**: conceptualization; writing—original draft; writing—review and editing; project administration. **Maurya Joshi**: supervision; validation; visualization. **Mohamed A. Omar**: methodology; software.

## CONFLICT OF INTEREST STATEMENT

The authors declare no conflict of interest.

## Data Availability

Data sharing is not applicable to this article as no new data were created or analyzed in this study.
